# The faces of God in America: Revealing religious diversity across people and politics

**DOI:** 10.1371/journal.pone.0198745

**Published:** 2018-06-11

**Authors:** Joshua Conrad Jackson, Neil Hester, Kurt Gray

**Affiliations:** University of North Carolina at Chapel Hill, Department of Psychology and Neuroscience, Chapel Hill, North Carolina, United States of America; Fordham University, UNITED STATES

## Abstract

Literature and art have long depicted God as a stern and elderly white man, but do people actually see Him this way? We use reverse correlation to understand how a representative sample of American Christians visualize the face of God, which we argue is indicative of how believers think about God’s mind. In contrast to historical depictions, Americans generally see God as young, Caucasian, and loving, but perceptions vary by believers’ political ideology and physical appearance. Liberals see God as relatively more feminine, more African American, and more loving than conservatives, who see God as older, more intelligent, and more powerful. All participants see God as similar to themselves on attractiveness, age, and, to a lesser extent, race. These differences are consistent with past research showing that people’s views of God are shaped by their group-based motivations and cognitive biases. Our results also speak to the broad scope of religious differences: even people of the same nationality and the same faith appear to think differently about God’s appearance.

## Introduction

**Fig 1:**
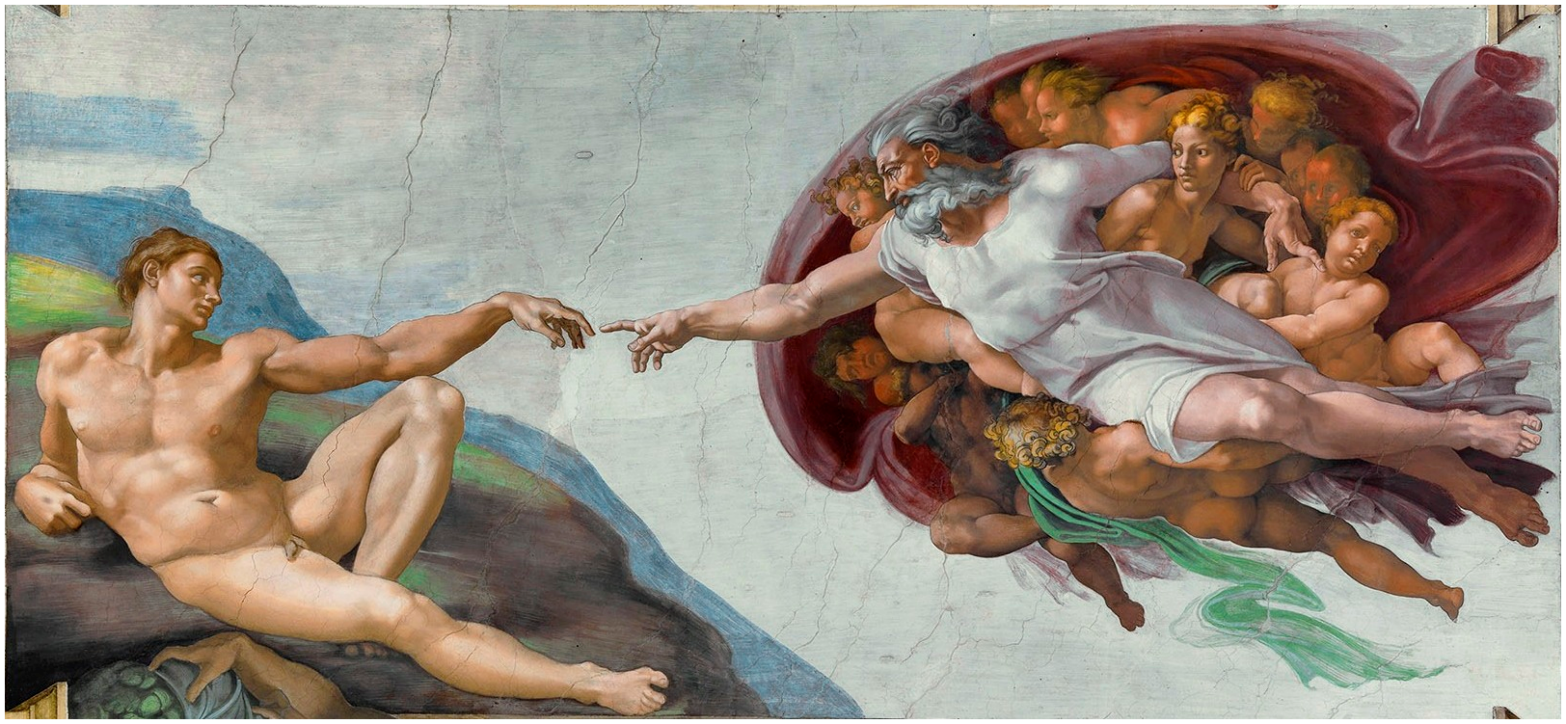
Michelangelo’s “*Creation of Adam*,” in which God (top right) is depicted as a stern, white-bearded man.

What does God look like? Although Exodus 33:20 states that “You cannot see my face, for no one may see me and live,” artists and writers have nevertheless depicted God’s likeness throughout history. From Michelangelo to Monty Python, popular illustrations have consistently shown God as an old and august white-bearded Caucasian man (see Fig 1). But do Christians really see God this way? To some people, God may seem younger, more feminine, and less Caucasian than popular culture suggests. In this paper, we use a new technique to reveal how American Christians see God’s face. As faces communicate both physical and psychological information, this measure also provides insight into how believers conceptualize God’s mind [1]. By showing how these perceptions vary within a religion, we can better understand the motivational and cognitive factors that shape people’s understanding of the divine.

### What’s in a face? The face of God as a measure of God’s mind

Despite depictions in stories, films, and renaissance-era paintings, people do not have definitive information about what God looks like. Genesis 1:27 describes man as created in God’s image, but other verses portray God as embodied as non-human (Exodus 3:2), or as not embodied at all (John 4:24). Consequently, since God’s appearance is not consistently described in scripture, people may draw from their assumptions about God’s “mind”—His temperament, personality, and capabilities [2–8]—when they visualize His face.

Past research on face perception supports the idea that when people visualize faces, these faces reflect assumptions about the minds of those who wear them. For example, when people visualize welfare recipients (versus non-recipients), they view them as having dull eyes to reflect their perceived lack of mental acuity [9], and when people visualize atheists (vs. non-atheists) they view them as having smaller eyes and narrow chins to reflect their perceived lack of honesty [10]. By extension, believers may see God’s face as smiling since God is typically viewed as all-loving and may also see Him as appearing powerful since God is viewed as almighty [11–12].

God’s face may be an especially useful measure of God’s mind because face perception measures are less susceptible to social desirability concerns than are verbal reports. For example, people will seldom admit that they assume welfare recipients are black but will choose darker faces when asked which of two alternatives looks more like a welfare recipient [9]. Similarly, people will seldom admit that they think of God as possessing human qualities—something Barrett [13] has termed “theological correctness”—but many believers implicitly describe God in humanlike terms [14] and may therefore project humanlike mental qualities onto God’s face. The question is: Which qualities will be conveyed by the face of God?

### How do people perceive God’s mind? The roles of motivation and cognitive biases

If people project God’s mind onto His perceived face, what should God’s face look like? Views of God are certainly shaped by scripture—the Quran describes God differently than the Bible—but even people within a religion may see God differently. Indeed, Christians’ descriptors of God seldom overlap [11, 15] and religious scholars have argued that images of God are best seen as idiosyncratic across individuals rather than monolithic within religion or culture [16–17]. A large body of research has documented the within-religion factors that might influence people’s views of God [18], including learning during socialization [19–20], prayer [21], and transmission biases [22]. Much of this work indicates that psychological processes may play an important role in how people view God. In particular, historical and contemporary research strongly suggests that *motivations* and *cognitive biases* jointly shape how people conceptualize God’s mind.

#### Motivation

The role of individual motivation in religious belief is a common theme of 19^th^ and 20^th^ century philosophy. Freud [23] claimed that belief in gods “derives its strength from the fact that it falls in with our instinctual desires”—specifically the need to form an attachment with a powerful father figure. Becker [24] tied religion to the motivation to transcend death, writing that “man cannot endure his own littleness unless he can translate it into meaningfulness on the largest possible level.” Marx [25] viewed religion as the “sigh of the oppressed creature,” suggesting that it fulfilled the motivation for control and autonomy.

Contemporary psychological research has echoed these perspectives by identifying specific motivations that influence the way people view God’s mind. People who lack control in their lives tend to see God as more powerful and influential as a form of compensatory control [26]. People who feel threatened by intergroup conflict conceptualize God as more authoritarian and punitive, since this kind of God could better regulate a society at war ([27], see also [28] for a perspective on natural disasters and views of God). And people with a strong need for a secure attachment tend to view God as more loving to provide themselves with an attachment figure [29]. Together, these perspectives suggest that people ascribe traits to God that help fulfill salient motivations.

#### Cognitive biases

While early philosophers emphasized the importance of motivation in religious belief, early anthropologists emphasized the importance of cognitive biases. Tylor [30] suggested that animism—the belief that natural phenomena possess agency—arose from conflating dreams with reality. Muller [31] also emphasized cognitive conflation in his explanation of religious belief, tying it to hyperactive anthropomorphism. Accounts like these inspired the modern cognitive science of religion, which views early religious belief as an accidental byproduct of evolutionarily functional tendencies, such as sensitivity to intentionality and agents in one’s environment [32–34].

People’s views of God’s mind appear especially susceptible to egocentric bias—overestimating how much others are like the self [35]. Observations of religious egocentrism have a long history: the 6^th^ century philosopher Xenophanes wrote, “Yet if cattle or horses or lions had hands and could draw, and could sculpt like men, then the horses would draw their gods like horses, and cattle like cattle; and each they would shape bodies of gods in the likeness, each kind, of their own.” Yet recent studies find that people think even more egocentrically about God’s mind than other people’s minds, and that self-oriented regions of the brain show more activation when believers think about God than when they think about other people [36–37].

### The present research

We introduce a face-visualization approach to measuring God’s mind and validate this measure in a large sample of American Christians. These data not only reveal how people generally view the face of God, but importantly show how motivations and cognitive biases shape believers’ understandings of God’s mind.

Motivation was operationalized via participants’ self-reported conservatism. Compared to liberals, American conservatives are more motivated to maximize social regulation, emphasizing law enforcement [38–41] and authoritarian leadership [42]. By contrast, liberals are more motivated to maximize societal tolerance, emphasizing intergroup harmony [38] and social justice [40]. These contrasting motivations suggest that conservatives may visualize an older, sterner, and more masculine God who is better suited to safeguard social order, whereas liberals may visualize a younger, kinder, and more feminine God who is better suited to encourage social tolerance.

Cognitive bias was operationalized by egocentrism, and we measured participants’ gender, age, race (African American versus White), and self-reported attractiveness. If people think egocentrically about God, they should visualize the face of God as being relatively like themselves for each of these qualities. We considered these superficial qualities to be particularly interesting because they would show that people view God as like them even in seemingly unimportant ways.

This study had two phases. In the first, we generated images corresponding to how people visualized God’s face and also measured individual differences. In the second phase, we asked separate samples of hypothesis-blind participants to rate these images of God’s face on (a) age, (b) gender, (c) attractiveness, (d) race, (e) happiness, (f) wealth, (g) intelligence, (h) lovingness, and (i) power. We predicted that that these hypothesis-blind ratings would reveal that, compared to liberals, conservative participants visualized an older, more masculine, whiter, wealthier, less loving, and more powerful God. We also hypothesized that the hypothesis-blind ratings would show that participants visualized a God similar to them in age, gender, attractiveness, and race. We included ratings of happiness, wealth, and intelligence as exploratory measures, with no a priori hypotheses.

## Materials and methods

### Image generation phase

#### Participants

We recruited a sample of 511 American Christians (330 men, 181 women; *M*_age_ = 47.37, *SD* = 16.41) through Qualtrics panels. This sample contained participants from Southern (*N* = 153), Northeast (*N* = 124), Midwest (*N* = 143), and Western states (*N* = 91), and over-sampled African Americans (26% African American and 74% Caucasian) in order to test for racial differences. More details about this survey procedure and design are given in the S1 File.

#### A method for mapping God’s face: Reverse correlation

**Fig 2:**
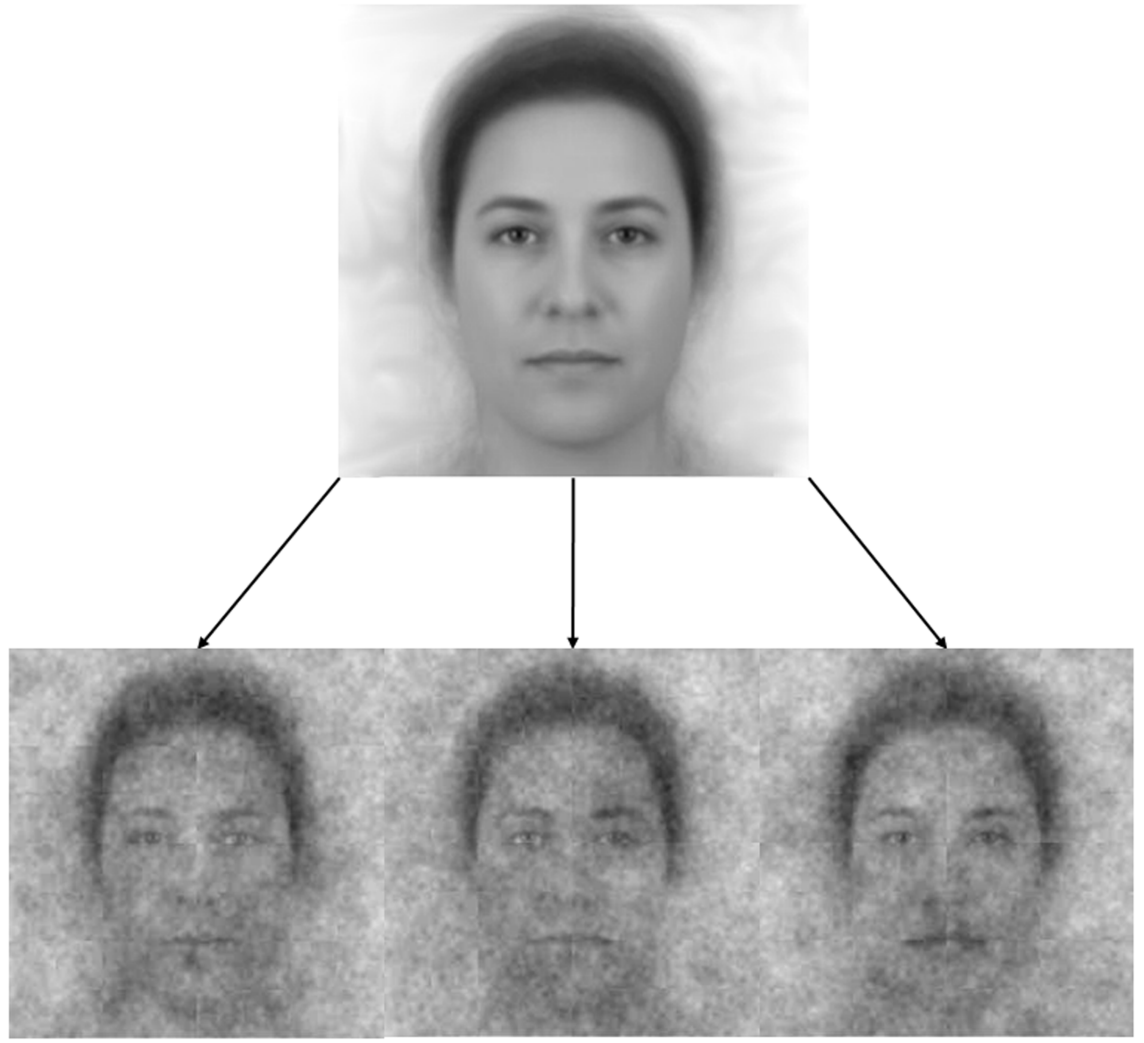
The base image (a composite of 50 faces that represent the collective demographics of the US population) and three of the 300 stimuli created by adding visual noise to the base image.

In order to measure people’s visualizations of God’s face, we used a nascent technique known as “reverse correlation” [43]. In reverse correlation, a face is repeatedly and randomly overlaid with visual noise to create many pairs of contrasting faces. Participants see these contrasting faces side-by-side on a computer screen and select the face from each pair that best matches their representation of a given target or category (e.g., “Which face looks more like a welfare recipient?” [9]). Collectively, these choices yield a complete face that represents how the owner’s mind is perceived. In our study, each participant viewed 300 face pairs, derived from adding visual noise to an “average” American face, which we created by combining 50 faces that represent the collective demographics of the US population in terms of age, race, and gender (see Fig 2). During the task, participants selected the face from each pair that better characterized how they imagined God to look.

#### Ethics statement

This study was approved by the ethics committee at the University of North Carolina, Chapel Hill (IRB #16–2747). All participants provided informed consent to participate in the study.

#### Self-report measures

After the reverse correlation procedure, all participants completed a demographics form, which included their age, gender, race, and their self-reported conservatism on a scale from 1 (“Very Liberal”) to 9 (“Very Conservative”).

### Image rating phase

#### Rating God’s face

After participants from the representative sample of American Christians generated their mental representations of God, we compiled these images to test our hypotheses. In particular, we averaged faces across (a) our entire sample of American Christians, (b) liberals and conservatives, and (c) men and women, (d) Caucasian and African American participants, (e) young and old participants, and (f) attractive and unattractive participants (self-reported).

These faces were then rated by three independent samples each containing 400 Mechanical Turk participants, with each sample evaluating a different set of stimuli. In our first sample, participants viewed the overall face of God side-by-side with the averaged faces that people *did not* select as looking like God (i.e., God’s “anti-face”). Participants then selected, on different screens in randomized order, which face was (a) older, (b) more African American, (c) more masculine, (d) more attractive, (e) happier, (f) wealthier, (g) more intelligent, (h) more loving, and (i) more powerful. Comparing these two images gives us an especially powerful comparison to reveal the perceived face of God.

In our second sample, participants completed the same procedure, while viewing the liberal’s face of God side-by-side with the conservative’s face of God. Finally, our third sample viewed (a) the young versus old people’s faces of God side-by-side and chose which face was younger, (b) the Caucasian versus African American people’s faces of God side-by-side and chose which face was more African American, (c) the men’s versus women’s faces of God side-by-side and chose which face was more feminine, and (d) the unattractive versus attractive people’s faces of God side-by-side and chose which face was more physically attractive. The left-right order of faces in all studies was counterbalanced to avoid confounding meaningful variance with a bias to simply pick faces on the right or left.

## Results

### The face of God across all American Christians

**Fig 3:**
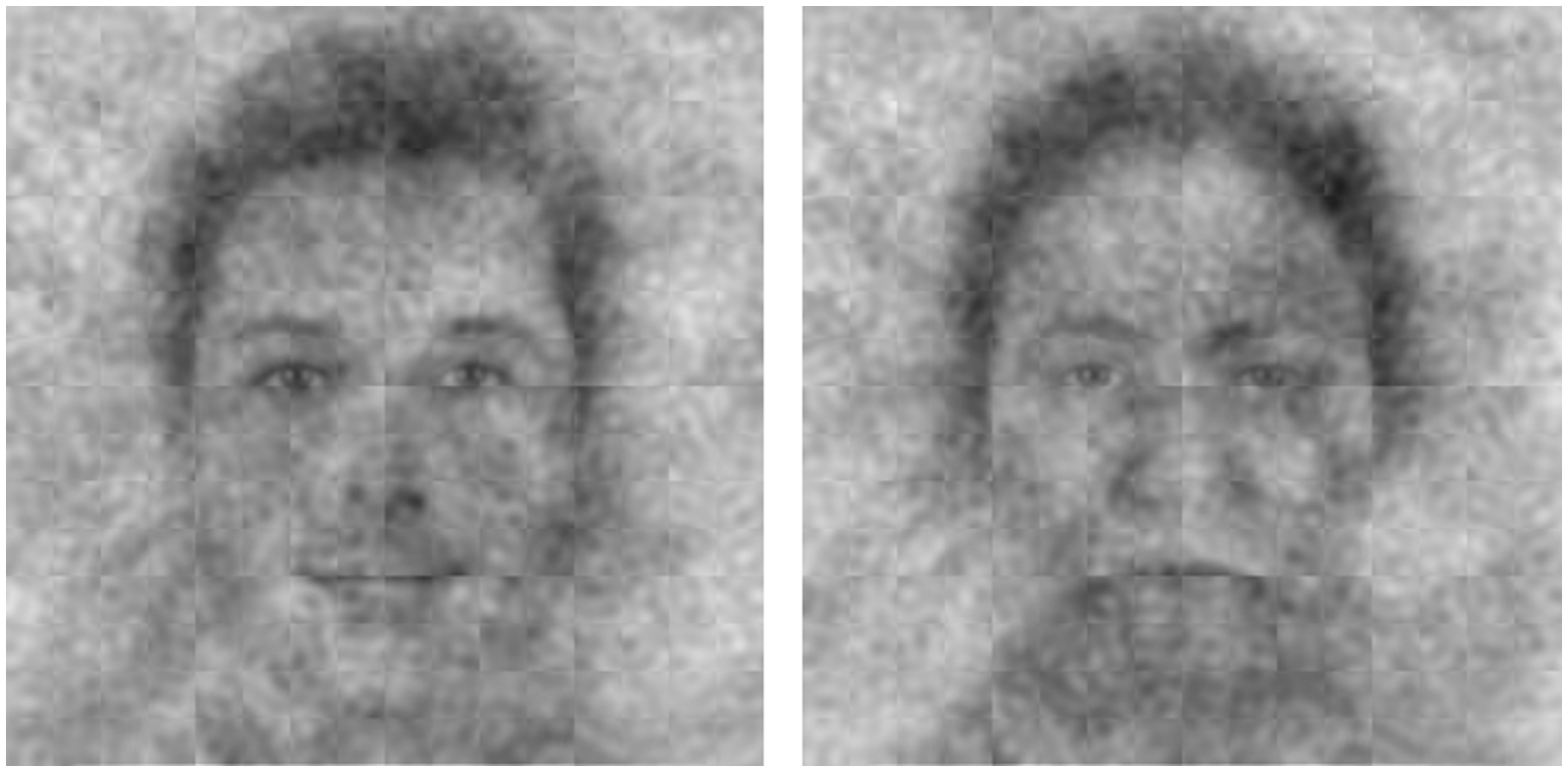
God’s perceived face (left) and anti-face (right) across American Christians.

What does God generally look like to American Christians? Participants saw God’s face as more masculine, Caucasian, attractive, intelligent, and loving compared to His anti-face, *t*s > 7.53, *p*s < .001 (see S1 Table for full statistics). See Fig 3. God’s face was also rated as significantly younger than the alternative composite, *t* = 31.83, *p* < .001, and as no more powerful, *t* = .47, *p* = .64, consistent with a general tendency for Americans to believe in a God who is more loving than stern [2]. Importantly, these differences were unbiased by the characteristics of the reverse correlation base image, since we compared faces that participants selected from those they did not select.

Together, these results help paint a picture of an American God who may not resemble scriptural or historical depictions. The face of the modern American God appeared kinder and more approachable than the God of the Sistine Chapel, perhaps reflecting different cultural concerns of the 16th century versus today. However, these general results should be interpreted with caution, since participants’ ratings may have been biased by their conceptualization of Jesus.

### The face of God across liberals and conservatives

**Fig 4:**
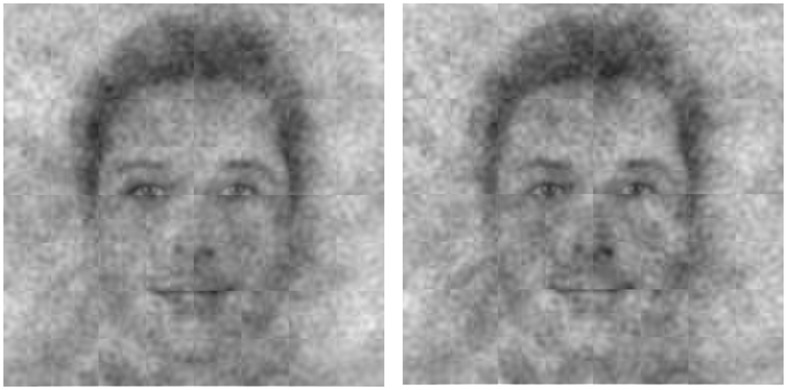
Aggregates of the images that liberal participants (left panel) and conservative participants (right panel) associated with how they viewed God.

Do liberals and conservatives see the face of God differently? To test this question, we generated composite images for those who self-identified in the bottom third of conservatism (i.e., liberals) versus the top third (i.e., conservatives). These images are shown in Fig 4. In our reverse correlation sample, conservative participants were more likely to be older, Caucasian, male, and more attractive, and so we covaried out these demographic factors when generating these composite faces in order to avoid confounding ideology and egocentrism.

**Fig 5:**
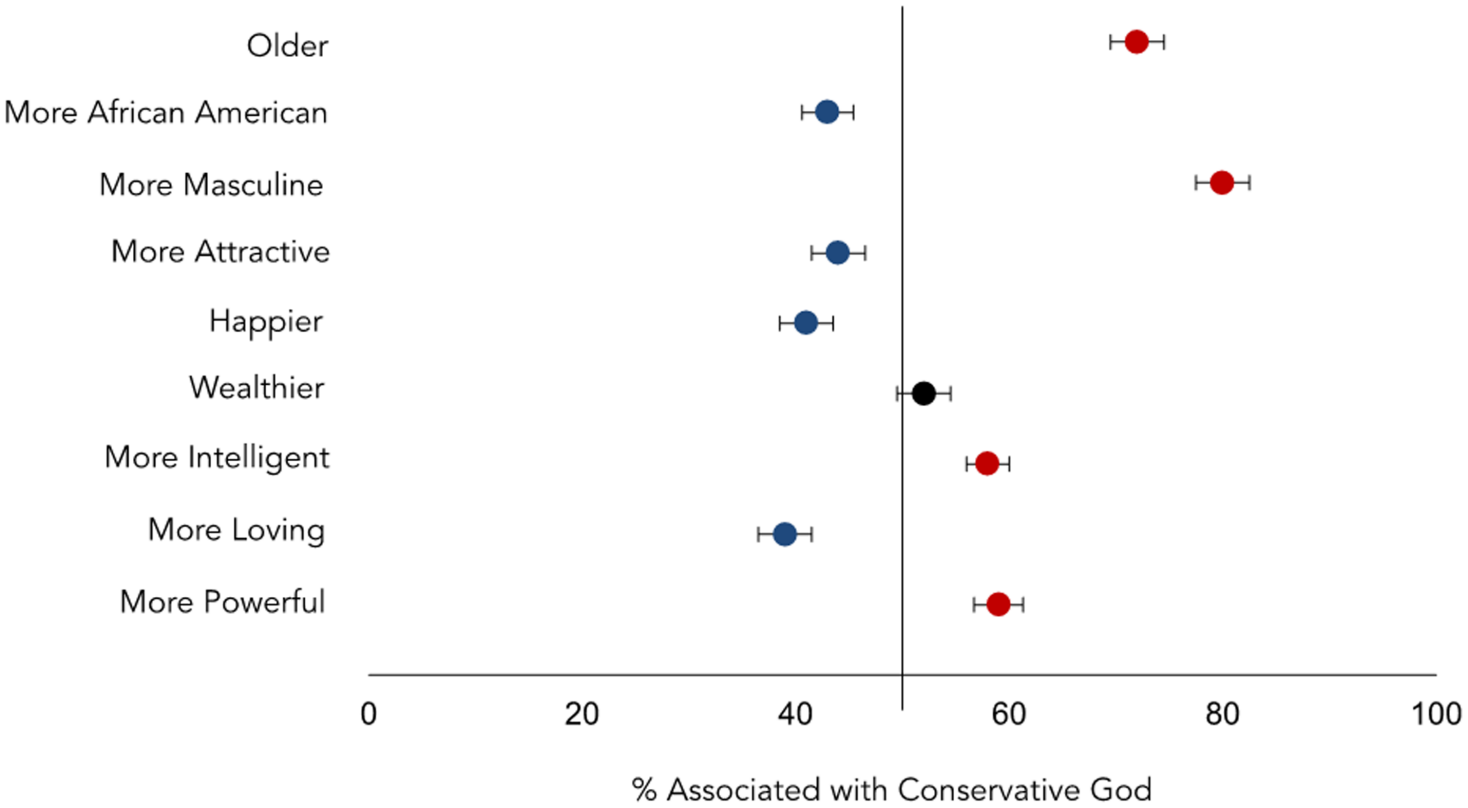
Liberals perceived God as more feminine, younger, more African American, more loving, and less powerful than conservatives. The horizontal axis represents the percentage that a specific feature was associated with a conservative (versus liberal) face. Error bars represent standard errors.

Independent ratings suggested that, as predicted, perceptions of God’s face are shaped by motivations tied to political orientation. The conservatives’ God was perceived as more masculine, older, more powerful, and wealthier than the liberals’ God, *t*s > 2.20, *p*s < .03, reflecting conservatives’ motivation for a God who enforces order. Conversely, liberals’ God was more African American and more loving than the conservatives’ God, *t*s > 3.49, *p*s < .002, reflecting their motivation for a God who encourages tolerance (see Fig 5; see S2 Table for full statistics). Conservatives visualized a God who was better-suited to meet their motivation for social order, while liberals visualized a God who was better-suited to meet their motivation for social tolerance.

### The egocentric face of God

**Fig 6:**
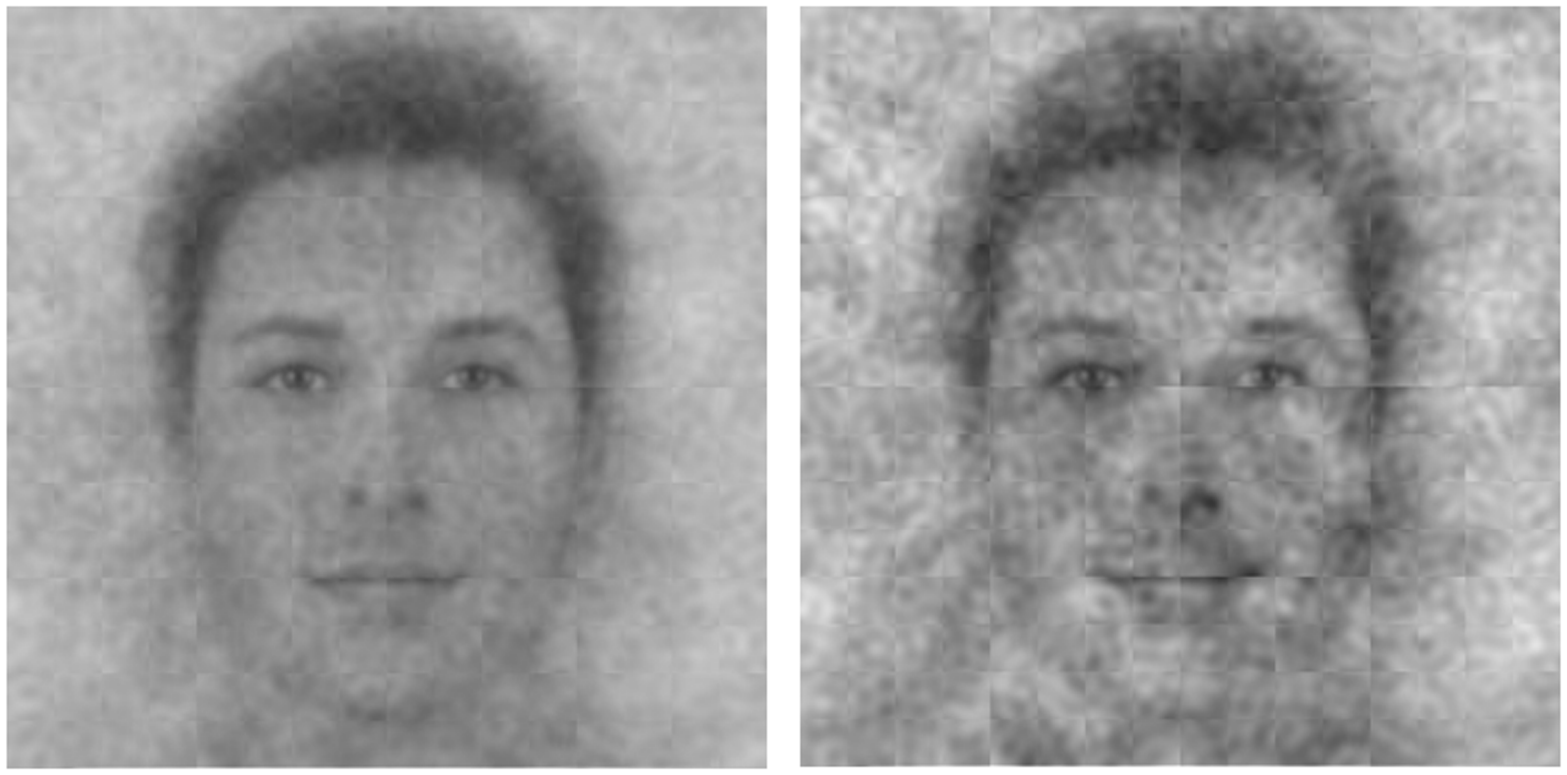
Aggregates of the images that young participants (left panel) and old participants (right panel) associated with how they viewed God.

Do people see a God who looks like them? Egocentrism suggests that people see the world and other people through the lens of the self. Perhaps the same is true with God, such that He shares not only people’s opinions, but also their facial features. We tested for the role of egocentrism in the perception of God by comparing God’s composite faces of (a) the youngest third of our sample with the oldest third of our sample (see Fig 6), (b) the least attractive third of our sample with the most attractive third of our sample (See S1 Fig), (c) African American participants with Caucasian participants (See S2 Fig), and (d) men versus women (See S3 Fig).

**Fig 7:**
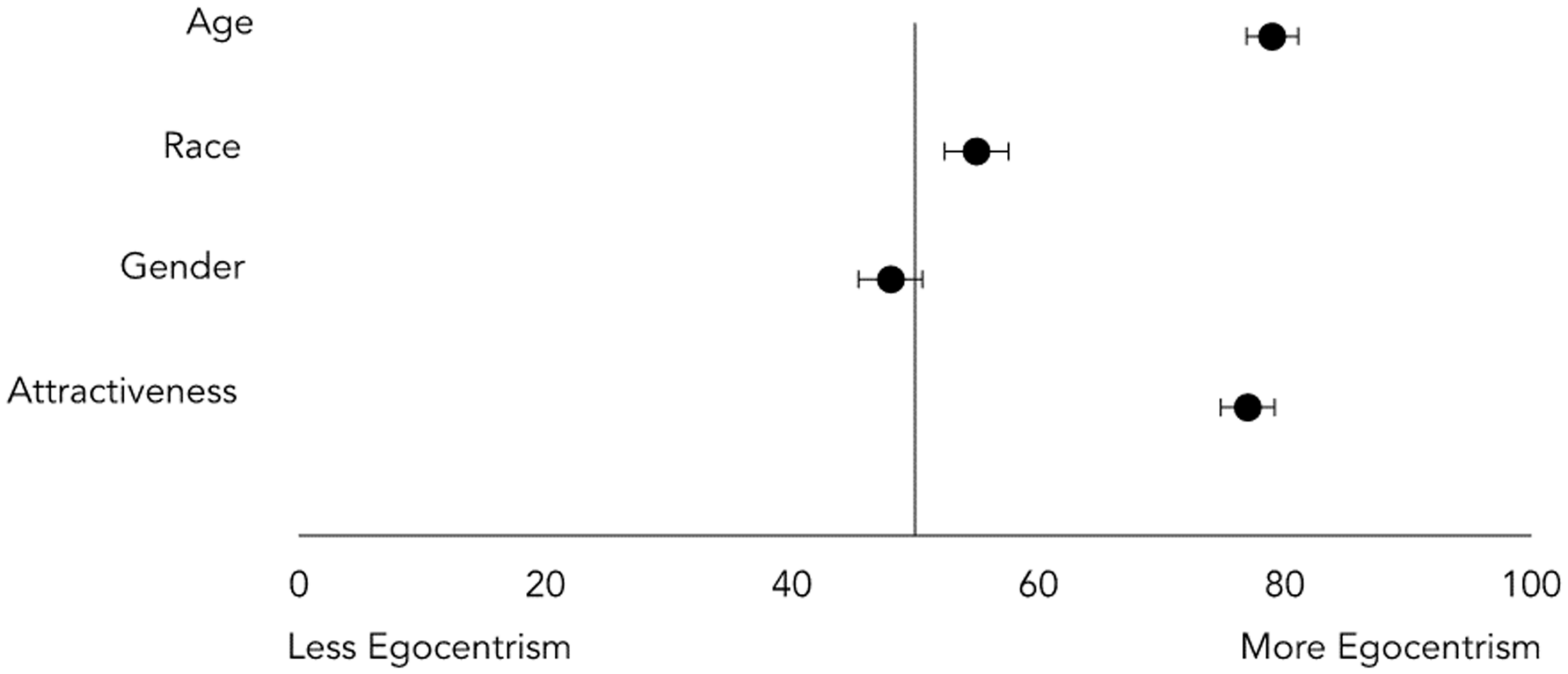
Egocentricity in perceptions of God. The perceived face of God was older for older people, more attractive for more attractive people, and marginally more African American for African Americans. The horizontal axis represents the percentage of trials in which a face was associated with its egocentric category. Bars represent standard errors.

Independent ratings suggest that, as predicted, perceptions of God’s face are shaped by egocentrism. Older participants saw an older God, *t*(377) = 13.96, *p* < .001, more attractive participants saw a more attractive God, *t*(378) = 12.33, *p* < .001, and African Americans saw a marginally more African American God, *t*(375) = 1.86, *p* = .06. Perceptions of God’s face did not vary across gender, *t*(377) = .93, *p* = .36; both men and women saw God as similarly male. These effects are shown in Fig 7. See S3 Table for full statistics.

## Discussion

We present a measure of God’s face that captures how believers think about God’s mind. In our sample of 511 American Christians, participants’ political conservatism and egocentrism shaped how they visualized God, consistent with past literature on how motivation and cognition influence views of God. Specifically, conservatives visualized a more powerful-looking God whereas liberals visualized a God who looked more loving. Participants also visualized a God who looked like them in terms of age, attractiveness, and, to a lesser extent, race; they did not, however, show egocentrism in terms of God’s gender.

These findings are striking in two respects. First, though many Christians claim that God’s appearance is unknowable [13], our sample of believers did appear to have stable representations of God’s face that included differentiable physical features (e.g. masculinity, youthfulness, and Whiteness) and psychological characteristics (e.g. lovingness). Second, even though American Christians ostensibly believe in the same God, people perceived Him in their own way, their perceptions reflecting their political ideologies and their own personal appearance.

### Caveats

Although our measure of the Christian God’s face is multidimensional, we only discuss nine dimensions of variance in this study: age, gender, attractiveness, race, perceived wealth, intelligence, happiness, lovingness, and powerfulness. These dimensions are not meant to be exhaustive and we encourage future researchers to test for other dimensions of variance using our data, which is publicly available at https://osf.io/y2rp3/files/. However, even this subset of dimensions demonstrates clear within-religion diversity in perceptions of God and help to explain how this diversity might emerge. If future researchers do re-analyze our stimuli, we recommend that they test hypotheses that involve multiple dimensions. Recent critiques of reverse correlation research have pointed out that, when testing single measures of variance and contrasting two faces, reverse correlation has a 50% error rate since any difference between faces could be detected at significance with enough raters. Our research is less susceptible to these critiques since it showed convergent support for hypotheses across multiple dimensions. Future research should similarly heed this potential weakness when testing hypotheses using reverse correlation.

Readers should bear in mind that this research was conducted on American Christians, meaning that our results cannot be generalized beyond this demographic. Our theoretical approach is informed by cross-cultural research and is intended to translate across religion and time; however, future work must investigate whether people’s views of gods are shaped by their own traits and motivations in the way that American Christians’ views appear to be. We anticipate that this will be an exciting area of future inquiry.

Our results were also limited by the fact that we did not collect denominational information. Could more conservative participants belong to denominations that emphasized different views of God? This seems unlikely because we observed no regional differences in God’s face (i.e., Northeast, South, Midwest, and West), even though Christian denominations vary substantially across regions. Nevertheless, future research could model denomination-level variance in views of God.

We also note that the results of reverse correlation are constrained by the base-image, and we used the representative American face. While we believe that image provided an appropriate referent for American Christians, it did not have prototypical features ascribed to the Christian God (e.g. facial hair), which may be interesting to use in follow-up studies. To test the generalizability of these results, future studies should also explore variance within other religious traditions that allow for visual depictions of gods.

Finally, some participants may have thought of Jesus during our reverse correlation procedure, which would explain why they visualized God’s face as more loving but not more powerful than the “anti-God” face. No participants admitted to using Jesus’s face when asked to freely report any difficulty with the study, but this may have occurred outside of their awareness, or else people may not have seen it as a difficulty. To some extent, the potential overlap between God and Jesus in our measure is inevitable because many Christians believe that God and Jesus are tightly bound together (i.e., the hypostatic union). And more generally, Jesus-God confluence is an artifact of any scale that measures views of God using anthropomorphic qualities. Nevertheless, this artifact does not undermine the validity of our central results—Christians’ views of religious agents are influenced by their political orientation and egocentrism.

### Implications

Our research has implications for theories and methods in the psychology of religion. By simultaneously modeling the impact of motivation and cognitive biases on perceptions of God, this research helps to synthesize two different literatures (see also [44]). Our model of within-religious diversity also ties religious belief to domain-general cognitive factors, consistent with the perspective that people construe God using many of the social cognitive processes by which they construe other people [45]. Our findings also suggest caution when using global measures (or primes) of religious belief, which assume that religious belief is a single construct. By revealing that God varies for each person along multiple dimensions, our data suggests that the link between religion and behavior may be nuanced. For example, as conservatives believe in a sterner God than liberals, conservatives may be relatively less likely to cheat after a religious prime [46].

Our findings also have implications for discussions of religion and public policy. Although the differences revealed here were subtle, they nevertheless revealed differences in elements of God (His appearance) that American Christians often assume that they agree on. These hidden disagreements speak to the fact that many religious conflicts are driven by the tension between believers assuming that God’s characteristics are universal while simultaneously seeing Him in their own way. Teaching people how perceptions of God vary even within religions may help increase religious tolerance.

### Conclusion

We began this paper with a question: What does God look like? Our results suggest that there may not be a single answer for all believers, even within the same religion. When believers think about God, they perceive a divine mind who is suited to meet their needs and who looks like them. Even though American Christians express belief in a universal God, their perceptions of His face are not universally similar.

## Supporting information

S1 File Description of study procedure. A detailed description of the measures, procedure, and recruitment strategy of our reverse correlation study.
(DOCX)

S1 Fig Attractive and unattractive people’s perceptions of God. Aggregates of the images that attractive people (left panel) and unattractive people (right panel) associated with how they viewed God.
(PDF)

S2 Fig Caucasians’ and African Americans’ perceptions of God. Aggregates of the images that Caucasian (left panel) and African American (right panel) participants associated with how they viewed God.
(PDF)

S3 Fig Women’s and men’s perceptions of God. Aggregates of the images that women (left panel) and men (right panel) associated with how they viewed God.
(PDF)

S1 Table Coefficients for ratings of God versus anti-God. Coefficients for ratings of God versus anti-God.
(DOCX)

S2 Table Coefficients for ratings of conservative versus liberal God. Coefficients for ratings of conservative versus liberal god.
(DOCX)

S3 Table Coefficients for egocentrism ratings. Coefficients for egocentrism ratings.
(DOCX)
