# Supplementary material for: The faces of God in America: Revealing religious diversity across people and politics
Source: PLoS One. 2018 Jun 11;13(6):e0198745. doi: 10.1371/journal.pone.0198745 (PMC5995373; doi:10.1371/journal.pone.0198745)
Supplement: S1 File — A detailed description of the measures, procedure, and recruitment strategy of our reverse correlation study. (DOCX) [file pone.0198745.s001.docx]

**Mapping God’s face: Further information**

**Participants**

Five hundred and eleven American Christians (330 men, 181 women; *M*_age_ = 47.37, *SD* = 16.41; 24% African American, 76% Caucasian) were recruited by Qualtrics panels to take part in a 30-minute study. We intentionally oversampled African American participants in order to test for effects of race with appropriate power.

**Procedure and Measures**

On the first page of the survey, participants reported their race, religion, and U.S. state. Participants were disqualified from the study (and were not included in our sample of 511) if they reported a non-Christian religion, or if they reported a race or geographic region that had reached its quota (e.g. if we had already gathered our requested quota of African American participants). If they qualified for the study, participants read instructions stating that, “in this study, you will see pairs of similar images depicting faces. For each pair, rate which one looks most like your understanding of God’s face. Of course, nobody knows what God really looks like, but please rate the face closest to how you imagine God looks.” Then, participants classified 300 images in terms of which image looked more like how they imagined God to look. Each pair of images was headed with the text “which face looks more like God.” Participants pressed the “f” key if they thought the leftmost face looked more like God and the “j” key if they thought the rightmost face looked more like God.

After classifying images, participants filled out two scales—Rosenberg’s self-esteem scale (12) and Glick and Fiske’s measure of hostile-benevolent sexism (13). Participants then indicated their gender, age, and how physically attractive they felt they were on a scale from 1 (very unattractive) to 10 (very attractive). Participants then reported on their primary language, their income bracket, their highest level of education, their standing on the political spectrum (from 1: very liberal to 7: very conservative), how frequently they attended religious services, how many times they had moved to a new town, their relationship status (single, in a relationship, married, widowed), their employment status (employed or unemployed), and their current levels of pleasantness and arousal (on scales anchored at 1 “very unpleasant/tired” to 9 “very pleasant/alert”). Many of these measures were purely exploratory and we have not yet analyzed their influence on how people imagined God’s appearance. After filling out these demographics measures, participants read our debriefing form, and ended the study.
