# Supplementary figures and images for: The faces of God in America: Revealing religious diversity across people and politics
Source: PLoS One. 2018 Jun 11;13(6):e0198745. doi: 10.1371/journal.pone.0198745 (PMC5995373; doi:10.1371/journal.pone.0198745)

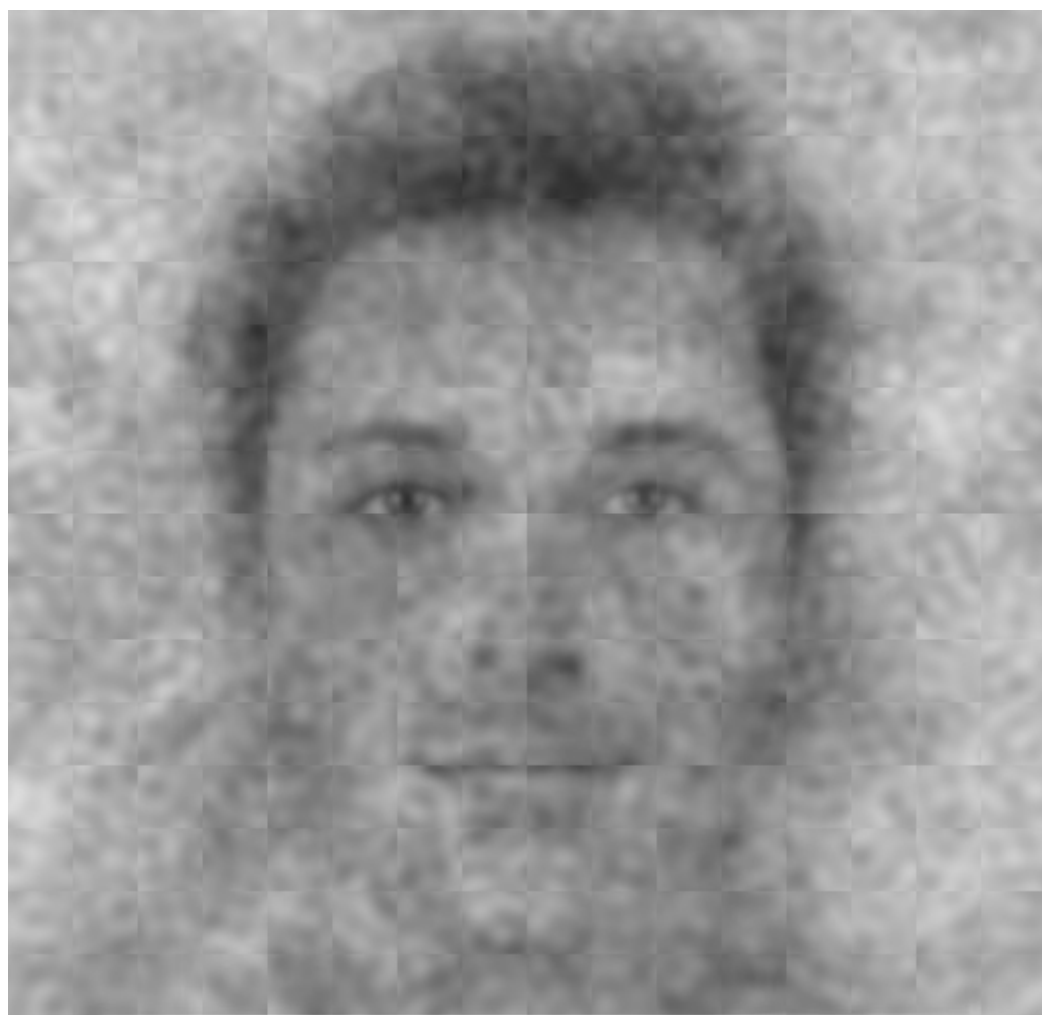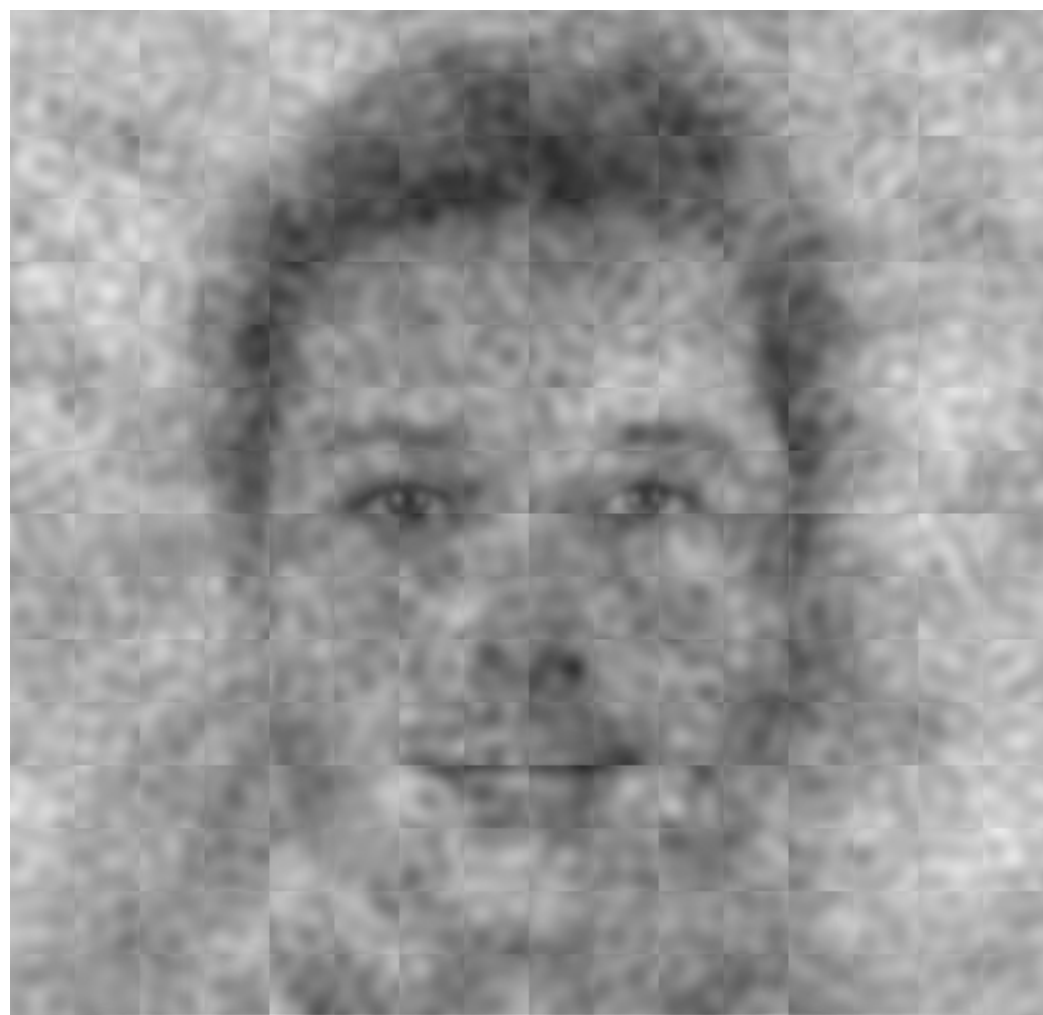

Supplement: S1 Fig — Aggregates of the images that attractive people (left panel) and unattractive people (right panel) associated with how they viewed God. (PDF) [file pone.0198745.s002.pdf]

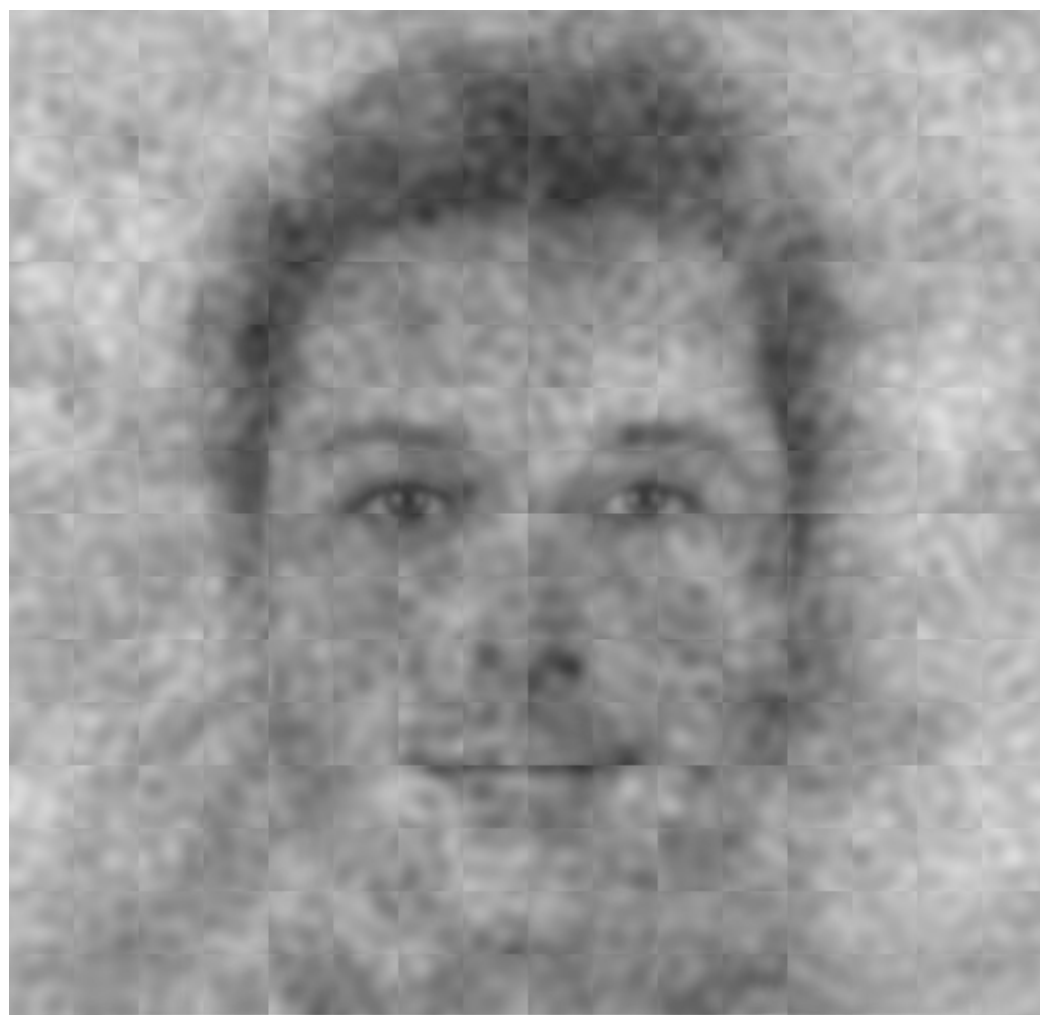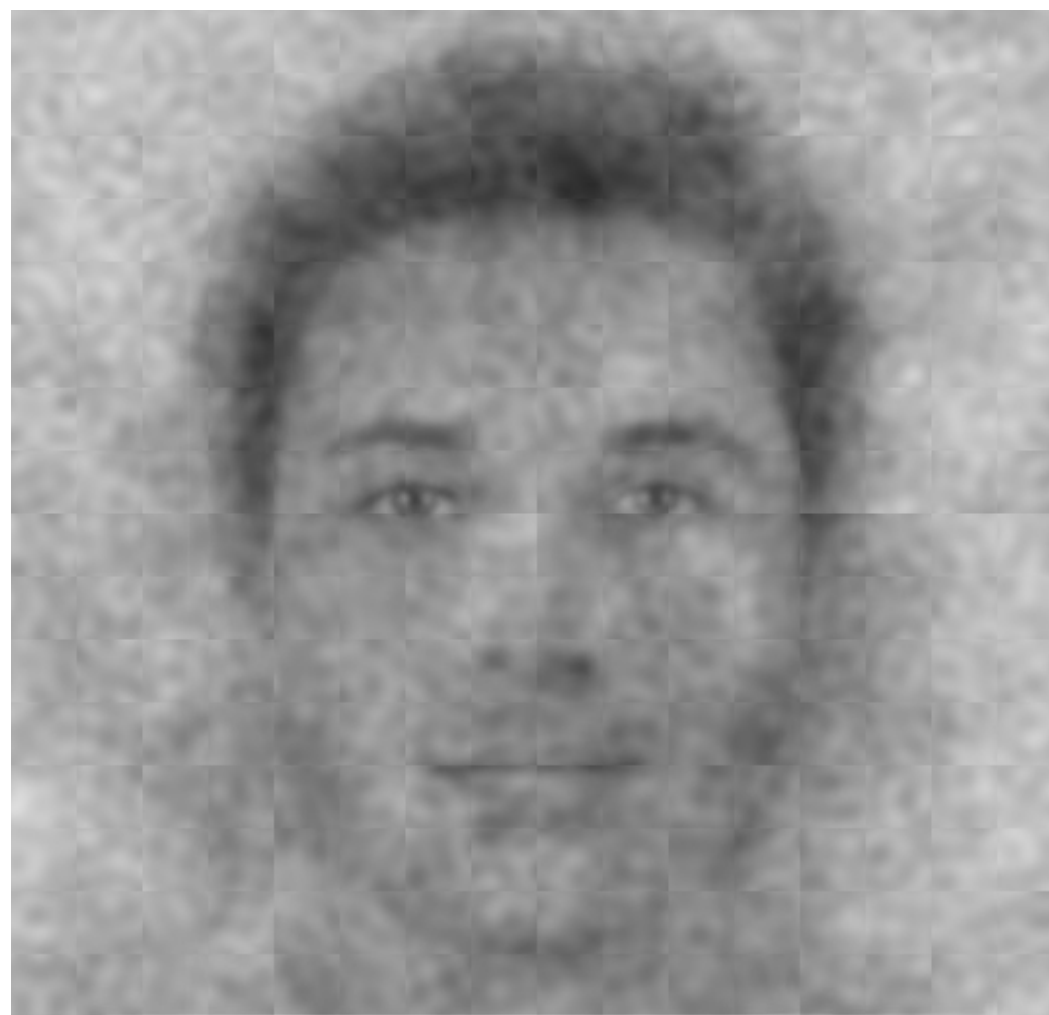

Supplement: S2 Fig — Aggregates of the images that Caucasian (left panel) and African American (right panel) participants associated with how they viewed God. (PDF) [file pone.0198745.s003.pdf]

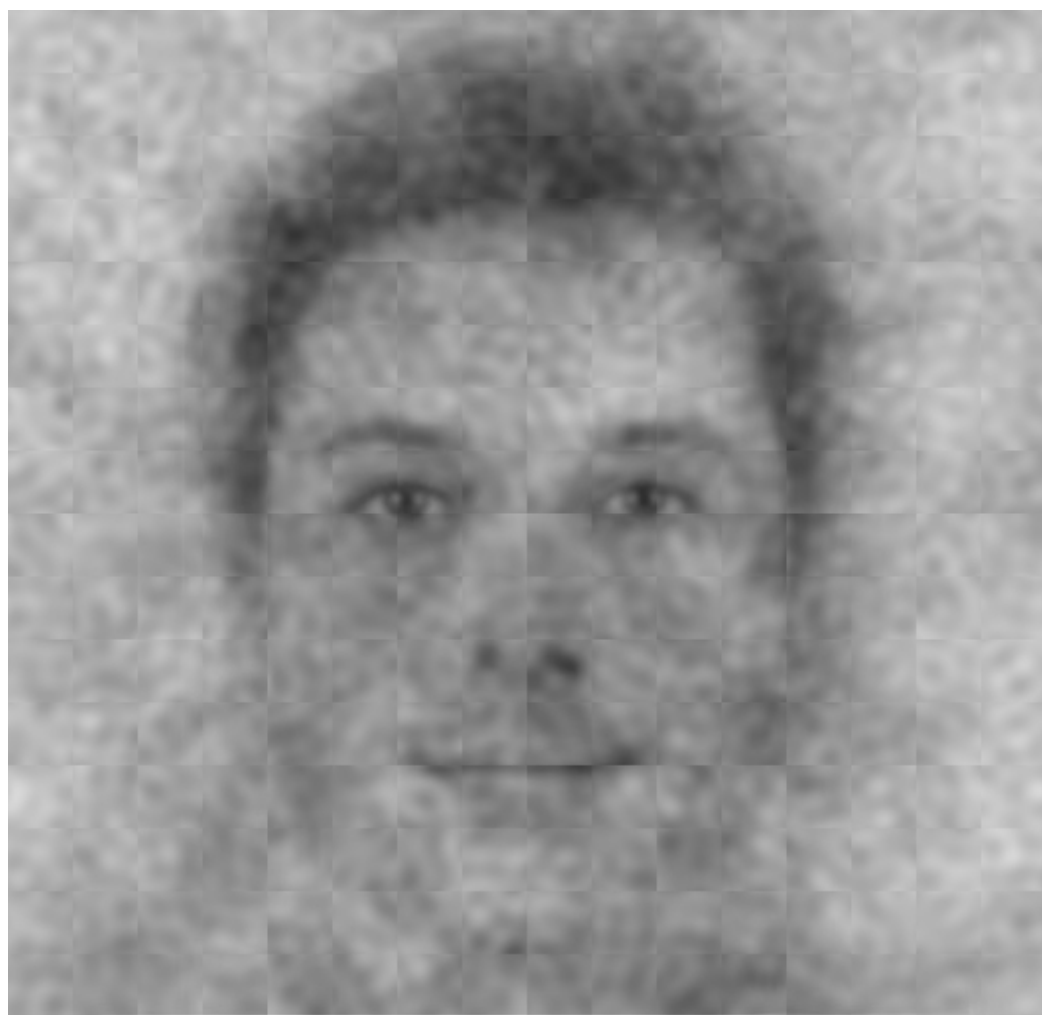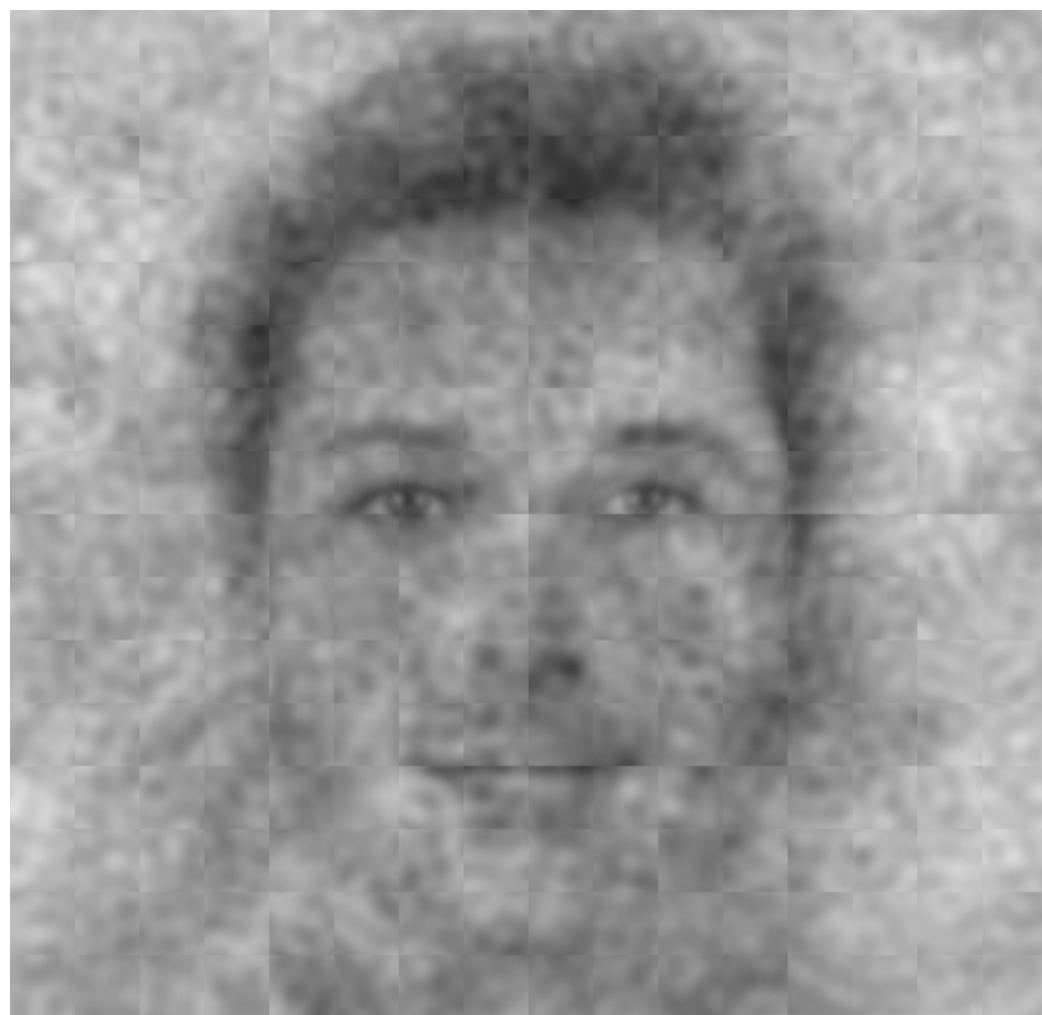

Supplement: S3 Fig — Aggregates of the images that women (left panel) and men (right panel) associated with how they viewed God. (PDF) [file pone.0198745.s004.pdf]
