# Supplementary material for: The faces of God in America: Revealing religious diversity across people and politics
Source: PLoS One. 2018 Jun 11;13(6):e0198745. doi: 10.1371/journal.pone.0198745 (PMC5995373; doi:10.1371/journal.pone.0198745)
Supplement: S1 Table — Coefficients for ratings of God versus anti-God. (DOCX) [file pone.0198745.s005.docx]

| **S1 Table.** Coefficients for rating of God versus anti-God | | | | | |
| --- | --- | --- | --- | --- | --- |
| Variable | *t* | *df* | *p* (2-tailed) | Lower 95% | Upper 95% |
| Older | -31.83 | 372 | < .001 | -45.40 | -40.12 |
| African American | -19.70 | 371 | < .001 | -39.32 | -32.18 |
| Masculine | 7.54 | 373 | < .001 | 13.44 | 22.92 |
| Attractive | 53.94 | 374 | < .001 | 45.35 | 48.78 |
| Happy | 59.98 | 373 | < .001 | 46.03 | 49.15 |
| Wealthy | 23.48 | 370 | < .001 | 35.44 | 41.92 |
| Intelligent | 21.19 | 374 | < .001 | 33.51 | 40.36 |
| Loving | 36.21 | 373 | < .001 | 41.72 | 46.51 |
| Powerful | .47 | 372 | .64 | -3.89 | 6.30 |

***Note.*** Positive *t-*values indicate association with composite God. Negative *t-*values indicate association with composite anti-God. Degrees of freedom are unequal across ratings because some raters did not rate all faces.
