# Supplementary material for: The faces of God in America: Revealing religious diversity across people and politics
Source: PLoS One. 2018 Jun 11;13(6):e0198745. doi: 10.1371/journal.pone.0198745 (PMC5995373; doi:10.1371/journal.pone.0198745)
Supplement: S2 Table — Coefficients for ratings of conservative versus liberal god. (DOCX) [file pone.0198745.s006.docx]

| **S2 Table.** Coefficients for ratings of conservative God versus liberal God | | | | | |
| --- | --- | --- | --- | --- | --- |
| Variable | *t* | *df* | *p* (2-tailed) | Lower 95% | Upper 95% |
| Older | 9.07 | 377 | < .001 | 25.75 | 16.58 |
| African American | -2.96 | 378 | .003 | -2.52 | -12.51 |
| Masculine | 14.68 | 377 | < .001 | 34.20 | 26.12 |
| Attractive | -2.23 | 376 | .03 | -.67 | -10.74 |
| Happy | -3.77 | 377 | < .001 | -4.55 | -14.50 |
| Wealthy | .51 | 379 | .61 | 6.36 | -3.73 |
| Intelligent | 3.17 | 378 | .002 | 13.04 | 3.06 |
| Loving | -4.54 | 377 | < .001 | -6.45 | -16,31 |
| Powerful | 3.49 | 378 | .001 | 13.82 | 3.86 |

***Note.*** Positive *t-*values indicate association with conservative composite God. Negative *t-*values indicate association with liberal composite God. Degrees of freedom are unequal across ratings because some raters did not rate all faces.
