# Supplementary material for: The faces of God in America: Revealing religious diversity across people and politics
Source: PLoS One. 2018 Jun 11;13(6):e0198745. doi: 10.1371/journal.pone.0198745 (PMC5995373; doi:10.1371/journal.pone.0198745)
Supplement: S3 Table — Coefficients for egocentrism ratings. (DOCX) [file pone.0198745.s007.docx]

| **S3 Table.** Coefficents for egocentrism ratings | | | | | |
| --- | --- | --- | --- | --- | --- |
| Variable | *t* | *df* | *p* (2-tailed) | Lower 95% | Upper 95% |
| Age | 13.96 | 378 | < .001 | 25.05 | 33.26 |
| Race | 1.86 | 375 | .06 | -.27 | 9.84 |
| Gender | -.93 | 377 | .36 | -2.68 | 7.44 |
| Attractiveness | 12.33 | 378 | < .001 | 22.51 | 31.05 |

***Note.*** Positive *t-*values indicate egocentrism. Degrees of freedom are unequal across ratings because some raters did not rate all faces.
